# Strategic Alliances in Global Health: Innovative Perspectives in the Era of Sustainable Development

**DOI:** 10.3390/healthcare12121198

**Published:** 2024-06-14

**Authors:** José Carlos Suarez-Herrera, Roberto Ariel Abeldaño Zúñiga, Lina Díaz-Castro

**Affiliations:** 1 Office of Research and Knowledge Transfer, Mid-Atlantic University, 35017 Las Palmas de Gran Canaria, Spain; josecarlos.suarez@pdi.atlanticomedio.es; 2 Yhteiskuntadatatieteen Keskus, Valtiotieteellinen Tiedekunta, Helsingin Yliopisto, 00150 Helsinki, Finland; 3 Postgraduate Department, University of Sierra Sur, Oaxaca 70800, Mexico; 4 Direction of Epidemiological and Psychosocial Research, National Institute of Psychiatry Ramón de la Fuente Muñiz, México City 14370, Mexico; dralaindiaz.ld@gmail.com

**Keywords:** global health, sustainable development, strategic alliances, cross-sector collaboration, universal health systems

## Abstract

This article discusses current challenges in the field of global health and the World Health Organization’s (WHO) strategies to address them. It highlights the importance of measuring the health impacts of global recession and globalization and the need for human-centered approaches to sustainable development. Emphasis is placed on commitment to health equity and the use of strategic partnerships for health at global, national, and local levels. Improving the health and well-being of populations, as well as public health equity, are core principles of the 2030 Agenda for the Sustainable Development Goals (SDGs). These principles are expressed in SDG 3, which promotes universal access to health services and systems and recognizes global health as a basic human right. It highlights the importance of strategic partnerships to combat emerging health crises, improve public health indices, and address the burden of chronic disease. These partnerships are contemplated in SDG 17 and are manifested in different modalities, such as network governance, cross-sector collaboration, public–private partnership, and social participation. This diversity of alliances has played an important role in scaling up and strengthening universal health systems around the world, including in Latin America and the Caribbean. The text concludes by presenting the essential characteristics of these inter-organizational and inter-institutional alliances in the field of global health.

## 1. Introduction

“*We build too many walls and not enough bridges*”—Isaac Newton.

Given the complexity of contemporary global health problems, international public health policies advocated by the World Health Organization (WHO) progressively incorporate specific strategies, complementing those previously addressed in the Declaration of Alma Ata on Primary Health Care [1,2]. This is the case with global health programs such as “*Health for All in the 21st Century*” [3,4] or “*Healthy Cities*” [5,6].

These programs include assessing the health consequences of worldwide economic downturns and globalization, emphasizing human-centered approaches to sustainable development; demonstrating unwavering commitment to health equity principles; and fostering extensive strategic partnerships across global, national, and local contexts [7,8]. Thus, in the late 1990s and early 2000s, the importance of interconnection between multiple countries, actors, and sectors of intervention began to be understood, giving rise to the networking of new collective commitments and co-responsibility, an approach considered by many global strategists as the only way to meet the challenges of society [3,9,10].

In this context, the field of global health is positioned as an essential body of knowledge and action to respond to societal challenges, particularly concerning its basic principles: (1) global health is a multi-pronged approach to health improvement worldwide that is taught and carried out in research institutions; (2) it is an ethically oriented initiative guided by principles of justice; and (3) global health is a mode of governance that produces influence through problem identification, policy decision making, as well as allocation and sharing of resources across borders [11,12,13].

These strategies are now fundamental principles of the 2030 Agenda for the Sustainable Development Goals (SDGs). They are particularly embodied in SDG 3, which aims to “*ensure healthy lives and promote well-being for all at all ages*” and recognizes global health as a basic human right [14,15]. This recognition necessitates the launch of a series of strategic alliances to actively combat emerging health crises, achieve substantial improvements in public health indices, and effectively address the growing burden of chronic diseases [16].

These partnerships are further enshrined in SDG 17: “*Revitalize the global partnership for sustainable development*”. In the field of global health, different modalities of strategic alliances, such as network governance [17], cross-sector collaboration [18], public–private partnership [19], and social participation [20], have played an important role in scaling up and strengthening universal health systems in countries around the world, including those in Latin America and the Caribbean (LAC) [21,22,23,24].

LAC countries present an interesting case in the fight against the COVID-19 pandemic due to their swift and pre-emptive actions to protect their citizens and contain the spread of the virus. Despite struggling with high levels of contamination, largely due to the prevalence of the informal economy and limitations of health infrastructure and social protection systems, these governments enacted measures based on community participation and governance of urban green spaces to mitigate the crisis. [21,22,23,24]. These experiences from the LAC countries offer important lessons for other regions in managing a health crisis while also addressing socio-economic challenges.

The purpose of this article is to present the essential characteristics of these new forms of inter-organizational and inter-institutional alliances. These alliances constitute an essential axis in the creation and updating of the field of global health. They represent a shift in the way we approach health challenges, moving from isolated efforts to a more integrated and collaborative approach. By understanding and leveraging these alliances, we can work towards a healthier and more equitable world.

## 2. Methods

We conducted a qualitative study based on a systematized, interdisciplinary review. This type of study has been applied on several occasions to explore cross-sector collaboration in the field of Public Health and Healthcare Management [25,26,27].

Unlike systematic reviews, systematized reviews are not intended to be comprehensive [28]. This research aimed to develop a structured and interdisciplinary review of a very broadly defined literature base without pretending to analyze exhaustively everything published about this article.

### 2.1. Collect Data

A search was carried out using English and French databases, and a set of scientific journals classified by various topics related to this research was selected. These reference databases are PubMed, 2000+ [biomedical and health sciences]; Persée; Érudit—Base; Google Scholar [Advanced Scholar Search]; Cairn; and Revues.org. Each of these databases offers different search functionalities. The research focused mainly on the period 2000–2024. 

In all databases, the key search term “*Global Health*” was used for all fields. We then selected articles that addressed at least one of the three key themes identified in our purpose: “*Sustainable Development*”, “*Cross-Sector Collaboration*”, and “*Universal Health Systems*”. We treated these terms as non-exclusive labels that delimit the relevant literature. Therefore, we used several synonyms and related terms in a series of searches, including those that we knew from experience were likely to appear in disciplines and areas of knowledge other than global health (Public Health, Environmental Research, Sustainability).

To obtain the widest possible range of literature, we used the most inclusive search fields available for each database. For “*Sustainable Development*”, we focused on “Agenda 2030”, “Sustainable Development Goals “, “Sustainability”, and “Social Sustainability”. For “*Cross-Sector Collaboration*”, we searched for “Intersectoral Action”, “Cross-Sector Partnership”, “Public-Private Partnership”, “Strategic Alliances”, “Social Participation”, and “Collaborative Planning”. For “Universal Health Systems”, we focused on “Universal Health Coverage”, “Health Coverage for All”, “Universal Health Care”, and “Health Diplomacy”.

Following these guidelines, we found 2567 documents through search engines on academic research and key journals. Additionally, we found another 280 documents identified through a purposive scan. When possible, we considered the citation criteria of selected articles (identified under the heading “*This article has been cited by […] articles*”), as well as those related to the selected articles (identified under the “*Related Articles*” section. This helped us find 14 more articles. 

Documents were included if the following criteria were met:

- Published between 2000 and 2024.
- English text versions are available.
- Discussed at least one of the three key themes identified in the purpose of our article:
  ⚬ Cross-sector collaboration;
  ⚬ Sustainable development;
  ⚬ Universal health systems.

All documents were independently reviewed for inclusion by three researchers. Disagreements on which articles to include or exclude were reconciled as a team.

Altogether, the combined searches yielded 534 articles in total (including duplicates). We then removed duplicates and made an initial assessment of relevance by reading the abstracts. This led to the removal of a large proportion of the preselected articles. Following this, we selected the 76 most relevant articles, i.e., those that explicitly appeared to deal with “*Cross-Sector Collaboration*”, “*Sustainable Development*”, and/or “*Innovation*”, broadly defined through “*Global Health*”, as our corpus of literature to analyze.

### 2.2. Data Analysis

The resulting articles were subjected to an abstract screening process to review the relevance of each article (and associated keywords) to the object of study. Articles were included if they represented original research or a review of the literature on the topics cited and explicitly documented these concepts in the body of the manuscript. The government documents used in this article are listed in the Endnotes section.

To systematize the data, a pre-categorization was organized according to our theoretical model, and a matrix was drawn up containing the following information: title, year of publication, study objectives, and results. Our database was updated as the literature review progressed. We organized the references listed in this review plan to retain only the most relevant. For this purpose, we used ZOTERO (v6.0.36) reference management software. We created and managed a database for the categorization of selected scientific references, whether articles, chapters, books, or scientific websites. More concretely, this software made it possible to manually enter and automatically import references (from catalogs, databases, scientific journals, etc.) to integrate citations into the manuscript to generate a bibliography. In addition, we included books and grey literature (scientific reports, working papers, and government documents) that appropriately address the main concepts related to the theme addressed in this article.

To assess the articles that met the inclusion requirements, the research objectives and results were summarized. The argumentation of this article was structured in the light of the results provided by the selected articles and related to each of the stages of our theoretical framework. It discusses the strengths and challenges of the new Quebec health and social services reforms in a globalized context of a socio-health crisis and a post-pandemic period. These questions were configured as thematic categories and were intended to guide the discussion and the preparation of summaries.

### 2.3. Ethical Considerations

As this is a systematized literature review that does not involve the participation of human subjects, this study does not require the approval of the ethics committee of our academic institutions.

## 3. Results

**Figure 1:**
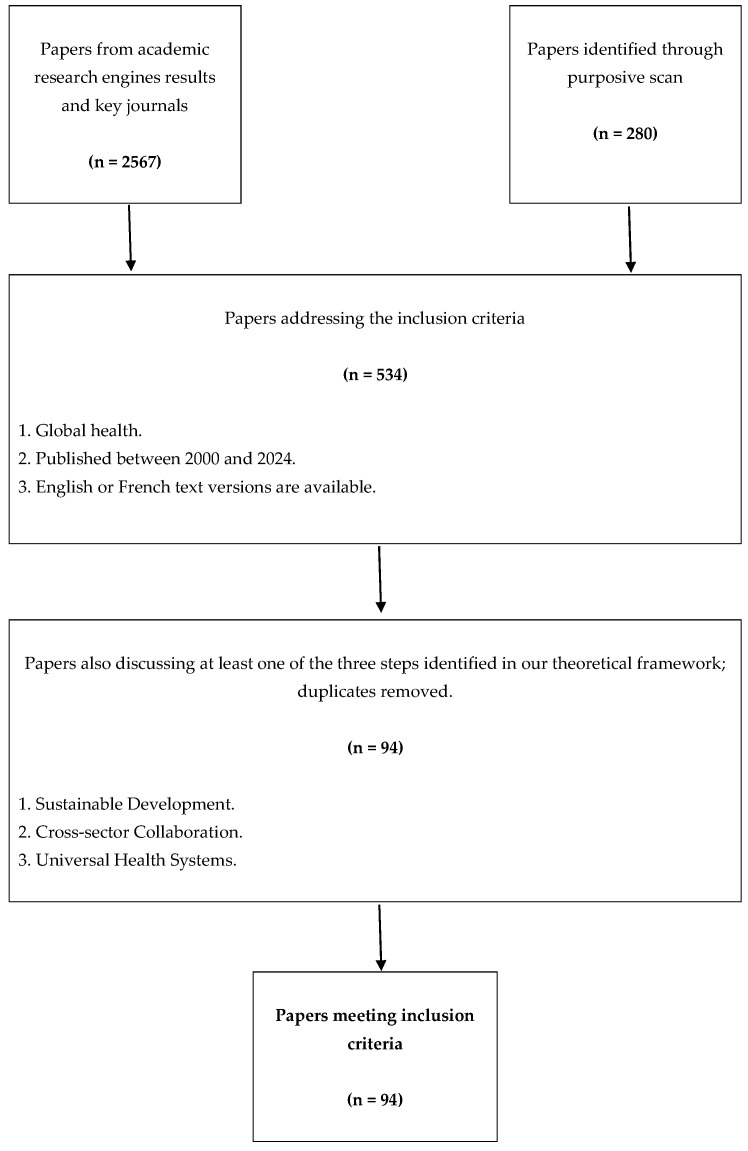
Flowchart of results.

The study made it possible to analyze 94 scientific articles published between 2000 and 2024 in publications related to the disciplines mentioned in the Section 2. The partial results of each stage of the article selection process are described in the flowchart shown in Figure 1.

In this section, we discuss the strategic alliances in the field of global health and put them in perspective with the key themes identified in our purpose. 

### 3.1. Global Health and the SDGs

In a neoliberal international context characterized by the globalization of public health and well-being challenges, it could be strongly argued that self-interest and collective altruism will converge in an increasingly complex and interdependent world [9]. The transnationalization of diseases and health risks requires global awareness, analysis, and action and indicates the need for more coordinated and inclusive forms of governance. Transnational action must be based on strong local and national conventions, but it also requires new forms of transnational collaboration to minimize risks and seize opportunities [10].

The development of nations is currently in a critical global decline, whether politically, financially, socially, or environmentally. Natural resources are being depleted, while indifference to the integrity of ecosystems—rooted in current political and trade practices—continues to grow [29]. In fact, despite the growing awareness on a global scale to the contrary, this indifference shown by some governments and numerous multinational financial institutions towards the long-term sustainability of resources (and our planet) and thus towards the health and well-being of future generations could be described as gratuitous [30] and, in some cases, negligence.

In this way, political leaders and health authorities responsible for public health and the development of territories are challenged to integrate sustainability issues (including global health governance, universal health systems, the promotion of social justice, and the protection of human rights) into global health strategies [31,32,33]. This incorporation makes a vital contribution to protecting both present and future generations and to reducing resource and health gaps between populations. In short, sustainability becomes a key element in the contribution of the field of global health to the structuring of a fairer and more responsible society [34,35]. 

#### 3.1.1. The 2030 Agenda for Sustainable Development

The creation and signing in 2015 of the 2030 Agenda for Sustainable Development testifies to the global and systemic scope of the challenges facing contemporary society. This initiative is structured around 17 SDGs that cover all the development issues that concern our global ecosystem, such as poverty, hunger, health, education, work, energy, water, violence, climate, and inequalities, among others [36].

The 2030 Agenda merges the United Nations Development Programme (UNDP) with the *Earth Summit Programme*, which is the term used to refer to the United Nations Conferences on Environment and Development, an exceptional type of international meeting between heads of state of all the countries of the world, to reach agreements on the environment, development, climate change, biodiversity, and other related issues. It is a universal approach, i.e., it applies to all countries, both North and South. In this regard, all the countries of our planet are called upon to adopt a socially responsible stance based on the sustainability of their territories. With its 17 SDGs and 169 targets (or sub-goals), the 2030 Agenda offers a detailed roadmap that covers virtually all the challenges of contemporary societies, including the health and well-being of populations.

#### 3.1.2. Global Health and Sustainable Development

The SDGs are a call to action by all countries to promote prosperity and protect the planet. They recognize that poverty eradication requires the implementation of a range of strategies that foster economic development and address growing social needs in the areas of education, health, social protection, and employment opportunities while combating climate change and promoting environmental protection. Now more than ever in the history of International Development Cooperation, the SDGs provide a critical framework for a collective and coordinated response to current global health challenges, such as climate change or epidemic and pandemic outbreaks [37,38,39].

SDG 3 of the 2030 Agenda, “*Ensure healthy lives and promote well-being for all at all ages*”, essentially includes 13 targets, which encompass the main health priorities and include the unfinished and expanded agenda of the Millennium Development Goals (MDGs), four targets to address non-communicable diseases, mental health, injury, and environmental issues, and four targets on the means of implementing these strategies.

In this context, the work of Sachs [14,40] highlights the critical role of improving health and well-being as both a remarkable achievement of our time and a formidable challenge for the next generation. The universal recognition of health as a basic human right and the commitment to ensuring healthy lives and promoting well-being for all underscore the centrality of global health and, by extension, Epidemiology, in achieving this ambitious goal. This perspective opens up new opportunities for the development of territories and their populations, emphasizing the potential of the field of global health as a driver of sustainable development.

The ethical dimension plays an important role in the framework of the 2030 Agenda. The SDGs, clearly more ambitious in scope than the MDGs, link development with sustainability to promote inclusion and reduce inequalities. According to some authors [15,41], health equity is a cross-cutting theme within an evidence-based conceptual framework that would help countries develop coherent action across all sectors and target areas of the SDGs. 

However, today, we are faced with the consequences of a development model that has neglected sustainability and equity and has been built on the intensive exploitation of territories and their populations [8,42]. The challenge posed here for the development of global health governance is essentially cross-cutting, affecting all categories of actors and all sectors of intervention. Sustainability and equity in health are aligned with the general principle of the SDGs and the implicit moral imperative of social justice that must accompany the set of collective responses to societal challenges [43].

In this context, WHO, as a standard-setting body with unprecedented constitutional powers, and despite considerable successes—such as the eradication of smallpox—faces numerous challenges in meeting health and human rights expectations [35]. By integrating the principles of social justice into the essential functions of universal health systems, some authors point out that existing human rights legislation would make it possible to establish the principles and foundations of global health governance beyond the political premises proposed by nations [44,45]. However, there is a governance gap between the human rights framework and international practices of global health and sustainable development policies. According to these authors, the current manifestations of the right to health in the 2030 Agenda are insufficient and superficial, as they do not explicitly link commitments or discourse on the right to health with the binding obligations of existing treaties in the framework of development cooperation on global health [46,47]. 

However, if we take the principles of global governance of health and social justice, the totality of the SDGs and their targets can be characterized as social determinants of health that, therefore, exert a great influence on the health of populations. For example, we could cite the elimination of hunger and poverty; the development of inclusive, equitable, and quality education; the fight for gender equality and the reduction in inequality within and between countries; or efforts to structure more inclusive, safe, and sustainable urban environments. Some authors point to the importance of addressing these social determinants of health through new forms of strategic partnerships involving the mobilization of all spheres of government, the involvement of economic institutions, and effective social participation [7,19]. 

Consequently, efforts to include the social determinants of health in the development of global health governance have begun to provide crucial information for the establishment of fairer and more sustainable strategic partnerships. These partnerships are based on collaboration—at regional, national, and international scales—between a wide range of organizations, including national governments, local authorities, international institutions, businesses, civil society organizations, foundations, philanthropists, social entrepreneurship investors, universities, and citizens. 

The SDGs offer a new opportunity for collaboration between global health and the rest of society and, hopefully, the impetus to move from words to action by considering health as a basic human right [14,48,49]. That said, to make these decisions—and to develop integrated action plans, strategies, and policies within the framework of global health governance—it is necessary to improve our understanding of the patterns of interaction and consultation among the actors involved [18,50,51].

### 3.2. Strategic Alliances in the Field of Global Health

It is worth noting the unifying potential of the SDGs—as well as that of the previous MDGs. Both the leaders of global health governance and the authorities responsible for universal health systems will have to demonstrate their capacity for a coordinated response, reform, and adaptability to the emerging challenges of contemporary societies [52]. Among the 17 SDGs, cooperation and strategic partnerships have been listed as key factors to respond innovatively and effectively to these challenges and achieve the targets set by the 2030 Agenda.

On the other hand, the recognition of global health as a basic human right requires the implementation of these strategic alliances and multilateral agreements to actively combat emerging health crises, achieve substantial improvements in the health indices of populations, and effectively address the growing burden of chronic diseases [7,16,19,30].

#### 3.2.1. Global Health and New Forms of Strategic Alliances

The configuration of strategic and multilateral partnerships is contemplated in SDG 17: “*Revitalize the global partnership for sustainable development*”. In the field of global health, we are witnessing the emergence of a series of concerted actions and interventions that play an important role in scaling up public health programs and strengthening universal health systems—based on sustainable development—in countries around the world, including those in Latin America and the Caribbean [7,53].

Such is the case of the “*Global Fund to Fight AIDS, Tuberculosis and Malaria*”, established in 2001 in the context of the AIDS pandemic and the MDGs. In this case, public–private partnerships played an important role in advancing health sciences and expanding and strengthening evidence-based public health activities in developing countries [19]. Such initiatives could serve as inspiration to fund research, development, and scale-up of interventions both in the field of health and in other areas of the SDGs of the 2030 Agenda.

Other authors also emphasize the importance of cross-sector partnerships that governments can establish with public health while also promoting the active and thoughtful participation of civil society. The sustainability of well-balanced cross-sectoral partnerships contributes to the successful performance of global health governance and, more importantly, to the good health and well-being of populations [54,55,56].

Studies on new forms of strategic partnerships to address priority determinants of health identify several key approaches, including stakeholder engagement and collaboration to collectively respond to current global health challenges [17,18,57]. Another key factor for the development of this type of alliance is collective learning [50], considered to be at the heart of effective cross-sector planning and initiatives, specifically because of its ability to constantly adapt strategies to changing circumstances and unforeseen situations affecting complex adaptive systems, such as universal health systems [58,59].

In the field of global health, based on a study of vector-borne diseases, Herdiana et al. [60] identify several factors influencing the effectiveness of cross-sector collaboration, such as the type of collaborative practices, responsible management of resources, quality of institutional relationships, governance modalities and shared vision. These authors point out that, despite its importance, very few studies have evaluated the degree to which cross-sectoral collaboration contributes precisely to the social impact of public health interventions and recommend further high-quality studies using indicators specific to this type of strategy. 

For their part, several groups of researchers have delved into this sense, evaluating the effects of strategic alliances in global health for research and education [61,62]. They identify several factors central to the success of global health programs, such as the constitution of a research steering committee; the development of a strategic action plan; securing institutional support and core funding; the development of collective leadership; monitoring and evaluation of interventions; systematic communication and coordination; and the creation of strategic networks between academic and institutional actors. 

In this sense, cross-sector networks are more likely to produce the desired effects when their members build a compelling operational framework on the strategic action plan (including a collective understanding of the problem, a consensus on solutions, and a set of plausible reasons for action), and when stakeholders succeed in building a political coalition involving influential actors beyond the health sector, a task that requires considering the full set of determinants of health, such as the SDGs of the 2030 Agenda [63,64]. 

Thus, according to Iedema et al. [63], within the framework of global health governance, health professionals and cross-sectoral actors should be involved in creating a multi-network structure. This structure requires deliberations and collaborations to be flexible and places network members in a position of “*strategic hybrids*”, allowing them to co-construct common sense and collective interest in response to current challenges [65,66]. 

Within the framework of the 2030 Agenda, the effectiveness of networks of actors and concerted actions could be assessed specifically in terms of sustainability. Some authors [67,68] note that, based on the SDGs, there are several challenges in shaping a sustainable cross-sectoral action network. The first is the *joint definition of the problem* (building consensus on the nature of the problem and how to address it). The second is *positioning* (representing the problem in a way that inspires external actors to act). The third is *coalition building* (forging sustainable partnerships with cross-sectoral actors). The fourth is *governance* (establishing an operational framework and a set of conventions that facilitate collective action). Research suggests that global health networks that effectively address these issues are more likely to gain support to address the conditions of concern.

Likewise, to achieve the SDGs of the 2030 Agenda, the range of collaborations should be expanded. Several authors show the growing and manifest importance of progressive civil society organizations as vital actors in achieving the necessary transformations [21,69]. These authors argue that a robust civil society can play several essential roles in the field of global health, such as building cross-sectoral coalitions beyond the health sector, ensuring human rights-based approaches, increasing the legitimacy of population health initiatives, improving accountability systems, and finally, an essential function that we develop in the next section—the strengthening of universal health systems.

#### 3.2.2. Integrated and Sustainable Universal Health Systems

A key aspect of the integrated and sustainable response to today’s public health challenges is the capacity of countries to strengthen universal health systems. The WHO considers that a universal health system comprises a set of organizations, people, and actions in a given territory with the primary intention of promoting, restoring, or maintaining the health of populations [70]. Universal health coverage, one of the health targets of SDG 3—essential to improve the health and well-being of populations—requires a significant increase in public investment in strengthening universal health systems [71,72].

This integrative vision goes beyond the logic of health action and extends to other sectors and systems that influence the health of populations (such as education or work) or that consider health to be an important part of their *raison d’être*, but with objectives that differ from those of the health system (such as development cooperation policies or strategies for the health system) to combat climate change. Thus, the ability to establish strategic alliances to strengthen universal health systems is essential to face catastrophic events, such as the economic crisis and the coronavirus (COVID-19) pandemic that are currently plaguing contemporary societies [53,73]. 

In other words, the contribution of universal health systems to the achievement of the SDGs would be strengthened thanks to the articulation of an inclusive network of cross-sector actors (at the global, regional, national, and local levels) in terms of principles and values, as well as in terms of vision and shared objectives that prioritize territories and their populations. Strong international cooperation is needed now more than ever to ensure that countries with the means to recover from crises strengthen their universal health systems and achieve the SDGs [32,74]. 

On the other hand, a series of concerted efforts are needed to achieve universal health coverage and sustainable financing of universal health systems, as well as to improve our response to global health challenges. As the COVID-19 pandemic has shown, universal, comprehensive, and sustainable health systems are better adapted and responsive to critical public health situations than non-critical ones, which treat health as a private service and not as a common good or a basic human right [75,76,77,78].

Perhaps the key to global health governance lies in a kind of strategic alliance between nations and international institutions based on “*health diplomacy*” [7,79,80], improving our response to health inequalities and promoting equity in global health [81]. The operationalization of this diplomatic practice requires the hard work of integrating the principles of global health into foreign policy deliberations and, therefore, into negotiations to create global health governance. Among the main areas of interest in the field of global health are security, trade, development, global public goods, human rights, and ethical/moral reasoning, which have been mobilized to promote the “*Health in All Policies*” program [82]. 

What is at stake here is the question of how to strengthen universal health systems through broad-based comprehensive and sustainable development, building on successes and promoting particularly beneficial transdisciplinary and cross-sector partnerships, in which synergies can be created between local and global actors, fostering “*strategic hybrids*” and improve the impact of global health interventions on territories and their populations [83].

## 4. Discussion

The authors cited in this article insist on the need for a renewed vision in the field of global health capable of adapting to the many destabilizing transitions in contemporary societies. From the point of view of our understanding of the processes underway, it is worth mentioning the need for a holistic, sustainable, and equitable approach necessary for the development of global health governance, integrated into the vision and purposes of the 2030 Agenda for Sustainable Development [45]. These initiatives need to go beyond the health approach to achieve a higher-order understanding of territories as complex cross-border, intersectoral, transdisciplinary, and multi-scale systems, with the integration of multiple actors, priorities, and solutions [84]. 

A good example of this is the role played by vaccines in the fight against the pandemic associated with COVID-19. Despite the positive impact the vaccines had in slowing the spread of COVID-19, progress in health technologies necessitates a comprehensive and meticulously coordinated process of collaborations across various sectors [85,86,87]. The epidemiological data reflecting the incidences and prevalences linked to the pandemic indicate that nations with superior coordination capabilities among their government bodies, health authorities, and civil society demonstrated more effective response rates [21,88]. This is in comparison to countries where there was a pronounced lack of coordination among these entities, despite some of these countries potentially having higher vaccination rates than those in the former group [89].

Our research underscores the confluence of these multifaceted interests within the complex terrain of political decision-making. The necessity for the participation of a variety of stakeholders in the decision-making process to guarantee holistic and efficacious global health governance is apparent [90]. Nevertheless, the difficulty resides in managing the conflicting interests of these entities while aiming for collective action. To address this predicament, our study proposes that the integration of a strategic alliance system may act as an enabling mechanism for achieving consensus among the involved parties.

In this sense, WHO emphasizes the importance of collaboration among 12 global health, development, and humanitarian agencies to expedite progress towards health-related SDG targets [35]. It presents a new approach to enhance collaboration and joint action, aiming to strategically use existing resources for improved health outcomes, underscoring that health is integral to sustainable development, linking SDG 3 to around 50 health-related targets across the SDGs.

A plausible proposal to achieve these ends would be to combine the principles of programs already implemented in this regard by the WHO, such as “*Healthy Municipalities and Communities*” [82,91], the “*Global Fund to Fight AIDS, Tuberculosis and Malaria*” [19], or “*Health in All Policies*” [92], with the SDGs of the 2030 Agenda. 

Here, numerous authors have confirmed the influence of knowledge transfer in improving health outcomes. Based on these studies, we emphasize the importance of education and knowledge transfer in improving the effectiveness of global health alliances [36,93,94,95].

The integration of cross-sector and multi-scale alliances in the governance of universal health systems, emphasizing social inequalities in health, would make it possible to address the socio-health priorities of territories and their populations, which vary considerably between countries, between territorial regions, between urban and rural districts, and along a timeline. Considering the infrastructural, political, economic, environmental, and socio-cultural determinants at different scales, these new forms of strategic alliance could lead, for example, to regional epidemiological and health forecasts that provide personalized messages to priority populations; integrated health interventions at the community and municipal level; more equitable access to nutritious food and green spaces; greater attention to the needs of the poor; and socio-cultural assets, spaces, and events that improve quality of life and human well-being [96].

Finally, despite some limits inherent to this type of research (impossibility of generalizing results, complexity of contextual variables, limitation of the study in time), we see the usefulness of systematized literature reviews in understanding the dynamics and importance of strategic partnerships in the field of global health: by conducting studies in low and/or middle-income countries, by studying the same case at different times, and even by finding common research problems for stakeholders with divergent interests.

## 5. Conclusions

By way of conclusion, we propose that the new field of global health be organized within the framework of the 2030 Agenda for Sustainable Development in the form of a “*transnational network of knowledge translation*”, that is, as a set of cross-sector and transdisciplinary actors that translate diverse knowledge and learn from each other [97,98]. Recognizing the cutting-edge role of knowledge management due to its innovative and transformative effects, the establishment of these networks is a key strategy for the development of integrated and sustainable universal health systems. Several authors [63,67,68,83] argue that collaborative efforts among cross-sector actors create innovative hybrid spaces within universal health systems. These spaces facilitate the co-production of knowledge and collective learning. They also enable rapid adaptation to the ever-changing environment while fostering collaboration between local and global stakeholders. Ultimately, these joint efforts lead to effective responses to current global health challenges. 

## Data Availability

The data supporting the findings of this review are the references themselves.
